# The architecture of visual narrative comprehension: the interaction of narrative structure and page layout in understanding comics

**DOI:** 10.3389/fpsyg.2014.00680

**Published:** 2014-07-01

**Authors:** Neil Cohn

**Affiliations:** Center for Research in Language, University of California San DiegoLa Jolla, CA, USA

**Keywords:** comics, visual language, narrative structure, visual narrative, page layouts, reading order

## Abstract

How do people make sense of the sequential images in visual narratives like comics? A growing literature of recent research has suggested that this comprehension involves the interaction of multiple systems: The creation of meaning across sequential images relies on a “narrative grammar” that packages conceptual information into categorical roles organized in hierarchic constituents. These images are encapsulated into panels arranged in the layout of a physical page. Finally, how panels frame information can impact both the narrative structure and page layout. Altogether, these systems operate in parallel to construct the Gestalt whole of comprehension of this visual language found in comics.

## Introduction

Comics have conveyed static drawn visual narratives for over a century, and growing research suggests that sequential images combined with text are an effective tool of communication and education (e.g., Nakazawa, 2005; Nalu and Bliss, 2011; Short et al., 2013), beyond just being entertainment. While theories about comics have been scattered in the humanities for several decades (for review, see Nöth, 1990; Cohn, 2012), only recently has scientific attention turned toward investigating just *how* readers comprehend complex graphic displays of sequential images. This growing literature of both theoretical and empirical research has established that extracting meaning from a comic page involves multiple interacting systems, analogous to the organization of a linguistic system (Cohn, 2013b): A *graphic structure* encodes the physical lines and shapes that compose the images, which construct meaningful expressions using a *lexicon* of stored graphic schemas. A *narrative structure* organizes these sequential images into a coherent message, while an *external compositional structure* arranges these panels across the physical layout of a page.

Altogether, these structures comprise the “visual language” that underlies comics, manga, graphic novels, and other visual narratives, which may also interface with text in larger multimodal interactions. Here, we focus on the systems most involved with sequential comprehension of a page: ***narrative structure*** and the ***external compositional structure***, which may be mediated by an ***attentional framing structure***.

KEY CONCEPT 1 **Narrative structure**

The system that packages meaning at a discourse level. This “visual narrative grammar” assigns categorical roles to images based on prototypical correspondences with a conceptual structure of meaning. These narrative units are organized into hierarchic constituents that allow for various types of embedding.

KEY CONCEPT 2 **External compositional structure**

The structure governing the organization of the physical layout of comic pages. These structures most often divide pages into horizontal and vertical constituents, though they also allow inset panels to be enclosed within a larger dominant panel, and Gestalt relations such as staggered, overlapping, and separated panels.

KEY CONCEPT 3 **Attentional framing structure**

The constraints on how conceptual information gets framed into panel units, determining how much content they contain. This has ramifications on how those images act in a narrative and how they are organized in a page layout (ECS).

## Visual narrative grammar

The question that has received the most attention regarding the visual language used in comics has been: How is meaning conveyed by a sequence of images? Early theories have focused on linear semantic changes between images (McCloud, 1993; Saraceni, 2003), consistent with prevailing theories of discourse structure (Halliday and Hasan, 1976). As a comprehender progresses through a discourse, they consistently monitor dimensions of time, characters, spatial location, and causality. Change in these dimensions requires an updating of the mental model being built from the complete understanding of the discourse (van Dijk and Kintsch, 1983; Zwaan and Radvansky, 1998), and inference for meaning left unseen (McCloud, 1993; Saraceni, 2003). Experiments have yet to examine these theories in the online comprehension of static visual narratives like comics, but research with film has confirmed that viewers can consciously identify these semantic shifts between individual film shots (Magliano et al., 2001; Zacks et al., 2009; Magliano and Zacks, 2011).

While empirical evidence supports that readers track semantic changes between linear image relationships, this approach alone cannot explain the comprehension of visual narratives. Problems with linear relationships first arose because of observations that non-adjacent panels sometimes necessitate long-distance connections in a sequence and panels often form meaningful groupings beyond linear relationships. Such intuitions aligned with empirical work showing that participants highly agree on where to divide sequential images into episodic constituents (Gernsbacher, 1985). The first alternative approach proposed a hierarchic model that created constituents based on changes of spatial viewpoint on a scene, changes between characters, or changes in time (Cohn, 2003, 2010). This approach revealed that linear relations between panels might be structurally ambiguous in ways explainable by underlying hierarchic structures (Cohn, 2003, 2013c). These basic groupings eventually gave way to observations that panels play functional roles in a sequence, similar to—yet somewhat different from—traditional narrative categories (e.g., Freytag, 1894; Mandler and Johnson, 1977). The resulting theory has been named “Visual Narrative Grammar” (Cohn, 2013c).

Visual Narrative Grammar (VNG) posits that, analogous to the way that sequential words take on grammatical roles that embed within a constituent structure in sentences, sequential images take on narrative roles that embed within a constituent structure in visual narratives (Cohn, 2013c). This is similar to previous “grammatical” approaches to narrative and discourse, such as the story grammars from the 1970s (e.g., Mandler and Johnson, 1977), yet these models differ in important ways (see Cohn, 2013c, for more details). It is important to stress that the comparison between narrative grammar and syntax is an analogy at the architectural level—images do not serve as nouns or verbs, and they convey information at a higher level than words (indeed, at a discourse level). Yet, narrative grammar uses a similar structural architecture as syntax, and these constructs are believed to operate in comprehension similar to the processing of syntactic representations. Whether these proposed similarities tie to common cognitive mechanisms is an active line of research.

VNG uses basic narrative categories to organize sequences: Establishers passively introduce the relationships between entities; Initials depict the start of an event or interaction; Peaks show a climax; and Releases depict a resolution or coda of events. While these categories form the core of a canonical narrative arc, other categories elaborate on a sequence, be it through additional narrative categories (Prolongations, Orienters), modification of the primary categories (Refiners, Perspective Shifts), or modification of the constituent structures (Conjunction) (Cohn, 2013b,c). Here, we will focus on the basic properties of VNG through an example sequence.

**Figure 1:**
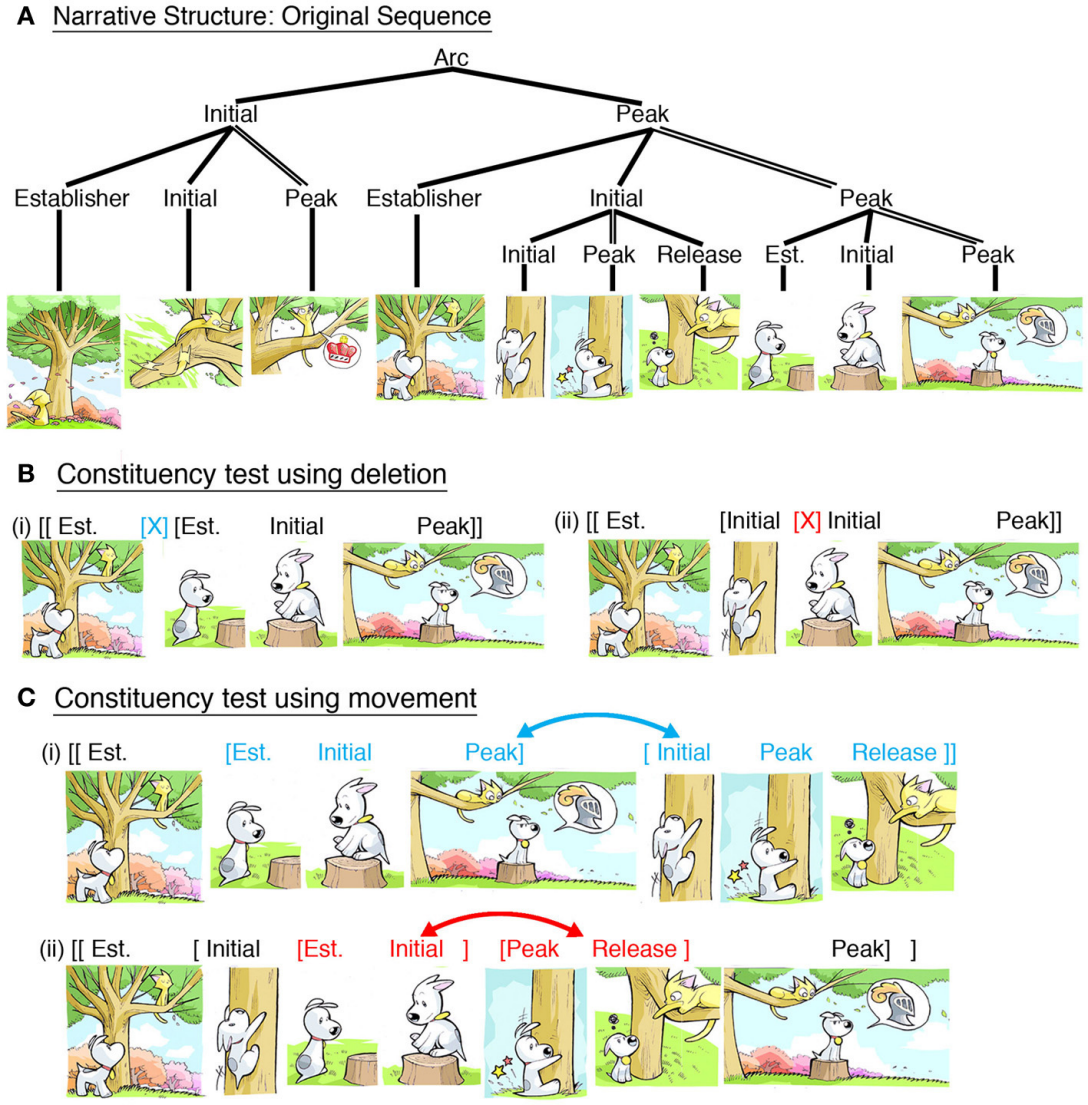
**(A)** Narrative structure of a sequence from the comic *Sinfest* (Ishida, 2008). Demonstration of constituent structures in this sequence comes from tests of deletion and movement: **(B)** Felicitous deletion of a whole constituent (i) and an infelicitous deletion of panels that cross the boundary between constituents (ii). **(C)** Felicitous rearrangement by moving the two substructures (i) and an infelicitous rearrangement of panels within those structures, crossing the constituent boundary. *Sinfest* and all characters © Tatsuya Ishida.

Consider Figure 1A, from the comic *Sinfest* (www.sinfest.net) by Tatsuya Ishida. An Establisher starts the sequence, passively introducing the relationship between the cat and the tree. The cat then begins his motion in the second panel, an Initial, climaxing as he reaches the tree branch in triumph, a Peak. Another Establisher then introduces the relationship between cat and dog, again with a passive state. The dog attempts to climb the tree (Initial), but he falls to the ground (Peak), resulting in the cat making fun of him (Release), a resolution to the dog's actions. The next panel Establishes a relationship between the dog and the stump, which he then hops onto (Initial) and assumes a protective role in a final climax (Peak).

Importantly, these categories do not just progress linearly, but also form groupings. The first three panels all depict the cat's climb, which forms an Initial to set up the second grouping of panels, which form a Peak, about the relationship between both cat and dog. An Establisher begins this second constituent by setting the new relationship, progressing to two substructures where the dog attempts to climb the tree (Initial), and then instead settles for sitting on a stump (Peak). Each constituent is motivated by its internal Peak, and the other panels support this primary panel. This primacy can be tested by omitting all panels except the Peaks, which should result in a paraphrase of the sequence. Hierarchic embedding allows sequences to have surface structures extending beyond the canonical narrative arc (*Establisher-Initial-Peak-Release*), though this ordering still is maintained within constituents. Thus, narrative categories recursively apply to both individual panels and groupings of panels.

Though VNG keeps the narrative structures separate from meaning, they maintain canonical correspondences between each other. For example, Initials prototypically depict preparatory actions (like the dog attempting to climb), while Peaks prototypically depict completed actions (like the cat reaching the top). However, narrative roles are not contingent upon such semantic correspondences and may have other mappings, such as the dog's *failure* to climb as a Peak. Narrative categories are influenced both by a panel's semantic *content* and its *context* within a sequence. This is analogous to how grammatical categories in language, like nouns and verbs, prototypically map to meaning, like objects and events, while ultimately being determined through their distribution in a sentence (Jackendoff, 1990).

Evidence for VNG comes from manipulating sequences in the same way that linguistics research manipulates sentences, such as using deletion or movement of panels or constituents (Cohn, 2013c). Consider Figure 1B, which depicts sequences where panels have been omitted from the second constituent of Figure 1A. Figure 1Bi deletes three panels that comprise the entire Initial constituent, resulting in a fully coherent sequence (note, omission of the whole first constituent is itself a successful deletion test). Figure 1Bii also omits three panels, but this deletion crosses the constituent boundary, therefore resulting in a more abrupt and awkward sequence. Next, Figure 1Ci rearranges the two substructures, resulting in a felicitous sequence (albeit with a less inspiring ending). However, rearranging panels that cross the constituent boundary, as in Figure 1Cii, results in a less felicitous sequence. Even though both rearrangements ostensibly take the sequence out of its original temporal order—and thus should both damage a sequence (Stein and Nezworski, 1978)—only (Figure 1Cii) results in a temporally awkward sequence. These diagnostics therefore offer support for the presence of constituent structures, and tests like these provide the basis for manipulations in experimental research, to which we now turn.

First, let's consider the experimental evidence that both content and context influence narrative categories, which has used tasks that highlight the distributional tendencies of panels (Cohn, 2014). One of these tasks asked participants to arrange four unordered panels into a coherent sequence, a technique used previously as a measure of “logical/sequential reasoning” in the WAIS test of non-verbal IQ (Kaufman and Lichtenberger, 2006). Though participants are highly accurate at these tasks, impaired reconstructions occur for deaf individuals who do not learn language until later in childhood (Mayberry, 1992) as well as Wernicke's and Broca's aphasics (Huber and Gleber, 1982; Fazio et al., 2009), while accuracy in this task correlates with age and experience reading comics (Nakazawa, 2005).

This “reconstruction task” was used to examine where narrative categories moved when they were misplaced in a sequence (Cohn, 2014). Initials and Peaks were moved around less than Establishers and Releases, and these latter categories appeared to fall in complementary distribution: panels that were originally coded as Releases were often moved to the front of a sequence to act as an Establisher, while panels originally coded as Establishers were moved to the ends of sequences. These sequence orders were among the most common of all sequence orders with misplaced panels. Further support for these complementary roles came from an additional task examining participants' self-paced viewing times to panels in sequences where two panels reversed positions within the sequence. No difference in viewing times arose between Establishers and Releases when their positions were reversed, either at the first or last position in the sequence. However, moving Peaks to the front or Initials to the end resulted in increased viewing times showing a cost of processing due to the “ungrammatical” sequences. These results suggested that panels acting as Establishers and Releases are more flexible in their positioning than those that act as Initials or Peaks.

Additional tasks in this study further showed the difference in importance between Initials/Peaks and Establishers/Releases. When participants were asked to arrange three of four panels and choose one to delete, they omitted Establishers and Releases far more often than Initials or Peaks. The reverse results occurred when participants guessed which panel was omitted from a sequence: elided Initials and Peaks were more accurately recognized as missing than Establishers and Releases. Such results expand on previous findings that participants have poor recall for omitted establishing shots from films (Kraft et al., 1991) or beginnings of verbal stories (Mandler and Johnson, 1977). Altogether, these complementary tasks show converging evidence that narrative categories have different distributional trends in a sequence—a finding that should not be feasible if comprehension only uses linear semantic relationships where panels play no particular roles.

When previous research has explicitly manipulated sequential images, the focus has remained on gross alterations of semantic congruity, such as findings that “scrambled” sequences of random images—the maximally “ungrammatical” sequences possible—are harder to understand than normally ordered visual narratives (Gernsbacher et al., 1990; Nagai et al., 2007). Additional research has examined sequential images using event-related potentials (ERPs), a measure of the electrical activity of the human brain allowing excellent functional and temporal resolution. In this work, anomalous final images of sequence were found to evoke larger “N400 effects” than congruous endings (West and Holcomb, 2002; Amoruso et al., 2013)—the N400 being a waveform associated with the access to semantic memory across domains, including language and visual images (Kutas and Hillyard, 1980; Kutas and Federmeier, 2011). Because viewers treat these incongruities as incomprehensible, it contrasts with popular notions that readers can incorporate any “non-sequitur” images into their understanding of sequential images (e.g., McCloud, 1993; Saraceni, 2003).

**Figure 2:**
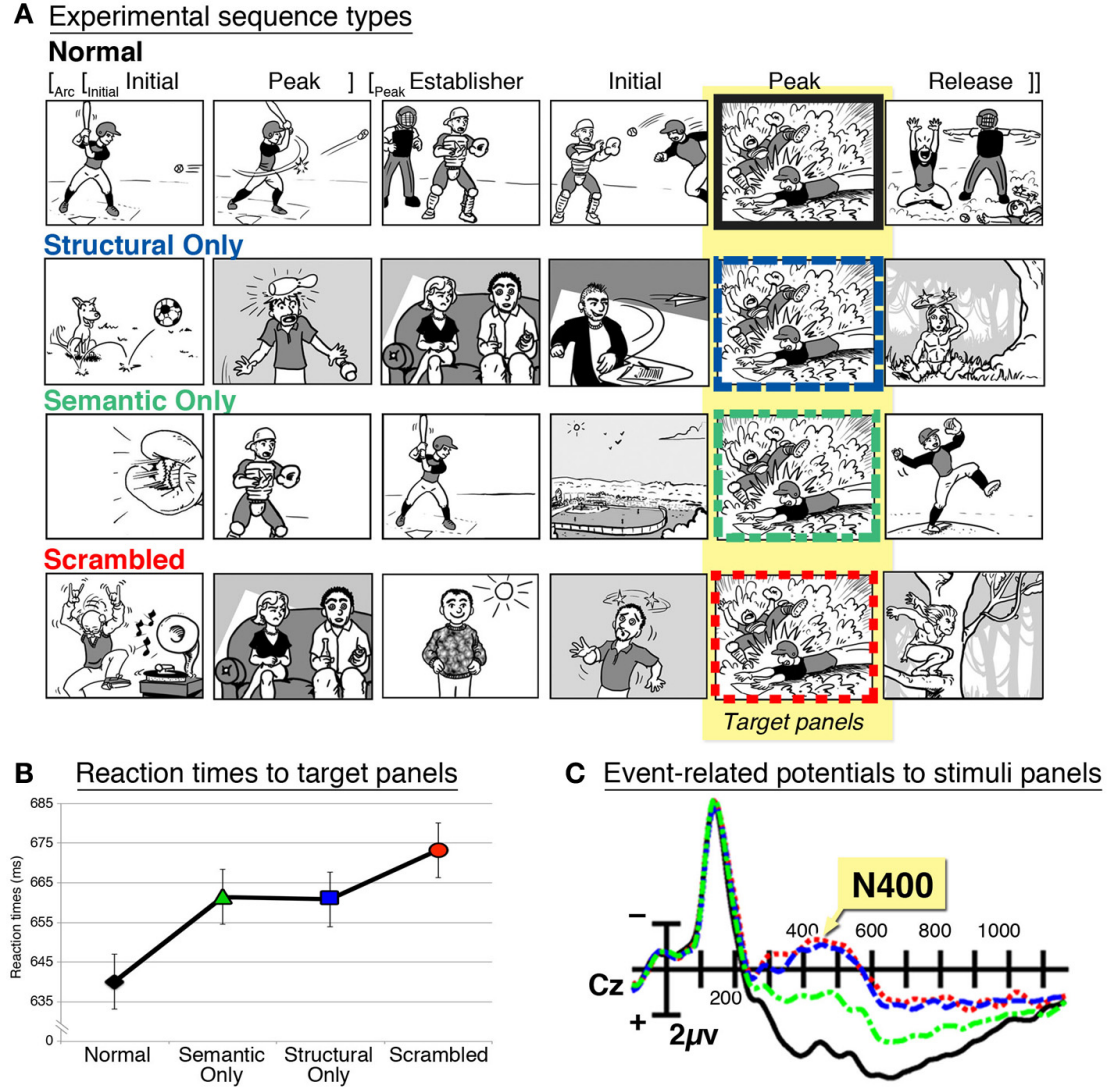
**(A)** Sequences manipulating narrative grammar, semantic associations, or both, which are similar to the stimuli in Cohn et al. (2012a). The narrative structure for this Normal sequence is shown, and it is matched by the Structural Only sequence. **(B)** Reaction times to target panels in these sequence types, and **(C)** event-related potentials showing an N400 effect to panels in these sequences.

These previous works have studied broad violations of meaning, but have not examined the balancing of narrative and meaning. These aims were undertaken in a study that replicated the research methods from psycholinguistics (Cohn et al., 2012a). Sequences were designed that had a felicitous narrative grammar, yet lacked semantic relationships between panels, analogous to Chomsky's (1965) famous sentence *Colorless green ideas sleep furiously*, which is grammatical yet lacks meaning. As depicted in Figure 2A, these “structural-only” sequences were contrasted with “normal” sequences which had both narrative and meaning, “scrambled” sequences which had neither, and “semantic-only” sequences which lacked a narrative structure but maintained semantic associations across panels (such as an overall theme). When participants monitored for target panels in these sequences, participants were fastest to respond to panels in normal sequences and slowest to those in scrambled sequences (Figure 2B). However, intermediate reaction times appeared to panels in structural-only and semantic-only sequences, suggesting that the presence of a narrative grammar or semantic associations gives an advantage to processing, though not as much as the presence of both. Such results parallel findings from classic psycholinguistics studies using target monitoring of words in analogously manipulated sentences (Marslen-Wilson and Tyler, 1980).

A second experiment in this study presented these same stimuli while recording ERPs. N400 effects were larger to panels from structural-only and scrambled sequences, intermediate to panels from semantic-only sequences, and the smallest to those from normal sequences (Figure 2C). These results suggest that the presence of narrative structure in structural-only sequences was not enough to attenuate the amplitude of the N400 effect, a waveform associated with *semantic* processing. Thus, while semantic information (including linear changes in coherence) clearly plays a role in the processing of sequential images, it does so in combination with a narrative grammar.

In addition, the amplitude of the N400 effect was attenuated across the ordinal position of normal sequences: the largest amplitudes appeared at the start of the sequence and became smaller as the sequence progressed. Because no such attenuation was found in other sequence types, this indicated both structure and meaning allowed for a build-up of meaning across a sequence. These findings again paralleled ERP results in analogous research of sentence processing (Van Petten and Kutas, 1991), and they also align with behavioral research showing that participants view images at the outset of a sequence slower than those later in the sequence (Gernsbacher, 1983; Cohn and Paczynski, 2013; Cohn, 2014). At the start of the sequence, readers may need more time to “lay a foundation” (Gernsbacher, 1985) of knowledge for the rest of the sequence (as in the function of an Establisher), which then allows for faster viewing (or attenuated N400 effects) as meaningful information accrues throughout the narrative.

Finally, though Cohn et al. (2012a) found no difference in the N400 effect between panels in structural-only and scrambled sequences, a negativity between these waveforms did appear in a localized left anterior region of the scalp. This distribution across the scalp differed distinctly from the more widespread negativity shown to the N400 effect, and was hypothesized to be similar to the left anterior negativity (LAN) effect evoked by violations of syntactic structure in sentences (Neville et al., 1991; Friederici et al., 1993). This left anterior effect was also correlated with a measure of participants' comic reading expertise—the more experience participants had, the larger the difference between these brain responses. Expertise effects like these are not unprecedented: the ability to accurately arrange images in a sequence and to infer missing panels correlates with both age and experience reading comics (Nakazawa, 2005; Nakazawa and Shwalb, 2012). Thus, not only do comprehenders utilize a narrative grammar in understanding sequential images, but such comprehension is modulated by their “fluency” in this visual language.

## External compositional structure

**Figure 3:**
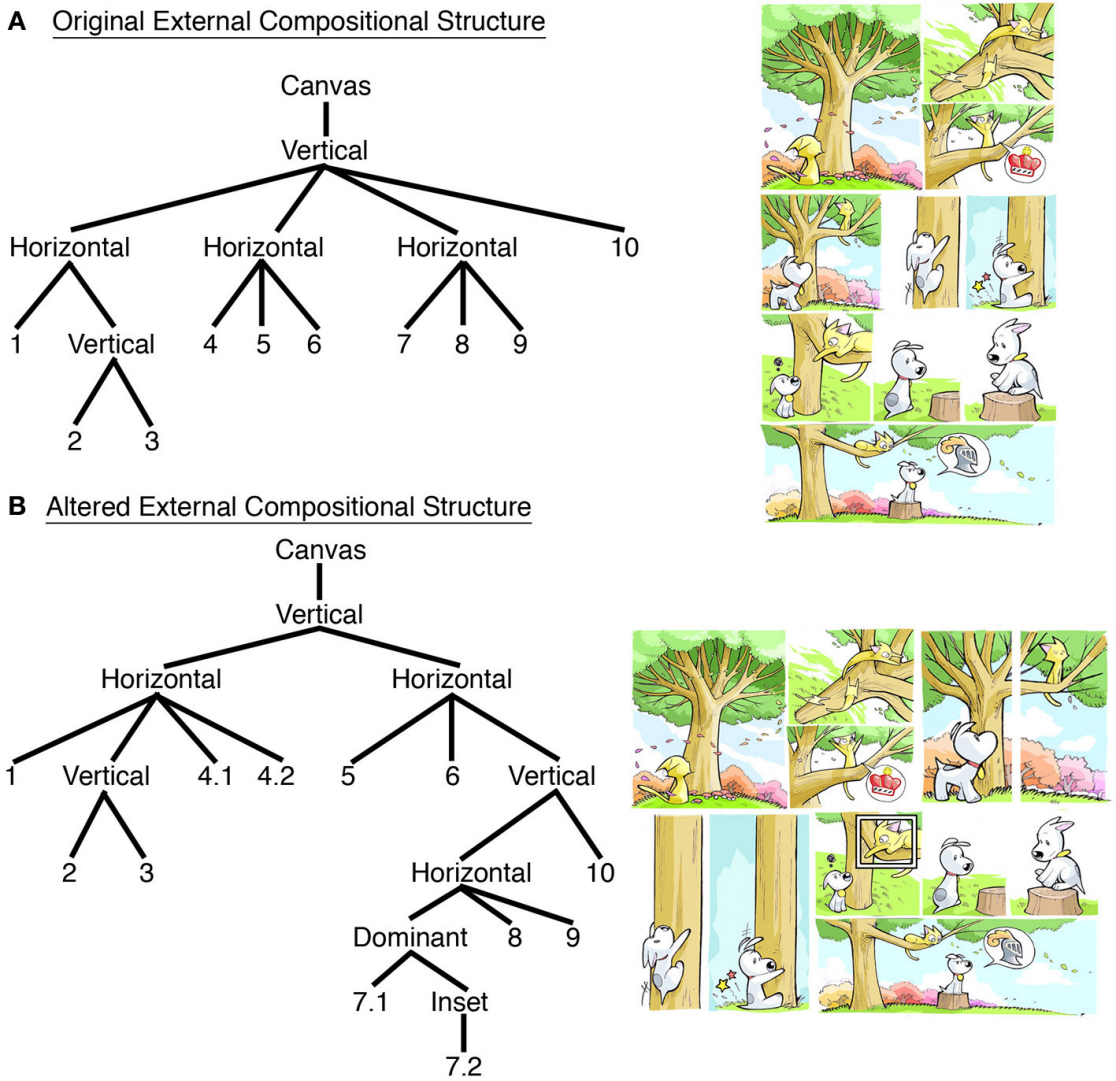
**(A)** Original page layout with its External Compositional Structure (ECS) diagrammed. **(B)** Alteration of page layout with resulting change to ECS. *Sinfest* and all characters © Tatsuya Ishida.

Separate from the content of a visual narrative, actual comics arrange panels physically on a page. Navigating this “external compositional structure” (ECS) of page layout cannot rely on the meaningful content of the panels since a single sequence can be arranged into numerous layouts with no effect on its meaning, as in Figure 3. This sort of rearrangement typically happens to comic strips when formatted for newspapers: they might appear as a horizontal strip, a vertical stack, or a four-panel grid. Unless these changes alter the actual order in which panels are read, then these alterations only impact the ECS, with no change in the conceptual/narrative structure. Moreover, data from eye-tracking experiments have shown that readers do not explore various potential pathways before progressing panel-by-panel (Nakazawa, 2002; Omori et al., 2004; Chiba et al., 2007), indicating that panel content does not provide the main motivation to their reading order (though an alternate order may be chosen if content confounds that intended order). Because of these reasons, ECS uses separate principles than those of the narrative/conceptual structures.

Typically, page layouts are thought to follow the left-to-right and down “Z-path” inherited by the alphabetic writing system (or the opposite, right-to-left, “reverse Z-path” of Japanese manga). However, pages often depart from this organization. Panels can be *separated* from each other, *overlapping* each other, or *staggered* next to each other so as to not create a continuous gutter between panels. In addition, *blockage* may occur when a long vertical panel appears to the right of vertically stacked panels, “blocking” a horizontal path of reading (as in the interaction between panels 2, 3, and 4.1 in Figure 3B).

These variations in layout were tested in a study where participants viewed comic pages devoid of content, and were asked to number the order that they would read these empty panels (Cohn, 2013a). Compared to the use of the Z-path in a canonical grid (0.95), viewers departed from using the Z-path only somewhat for staggering (0.89) or separation (0.71), but departed greatly for blockage (0.31). This effect for blockage was modulated by comic reading expertise: Participants with little or no experience reading comics were far more likely to use the Z-path than those with any experience at all. Nevertheless, eye-tracking data suggests that some readers skip over the vertically stacked panel in blockage layouts in favor of the horizontal Z-path order (Omori et al., 2004; Chiba et al., 2007). Thus, asymmetries may exist between preferences for a page's layout (ECS) and how people navigate that page, perhaps conditioned by their “fluency” in this visual language.

These experimental results suggested that several constraints factor into how readers navigate page layouts. A general strategy of ***Assemblage*** guides readers to seek to build units of structure that create coherent shapes in as smooth a reading path as possible (Cohn, 2013a). These preferences are: (1) grouped areas are preferred to non-grouped areas, (2) smooth paths are preferred to broken paths, (3) one should not jump over units, and (4) one should not leave “gaps” in reading. Thus, readers prefer to move down vertically in blockage paths rather than horizontally because they seek to create a whole grouping of contiguous panels without leaving a gap.

KEY CONCEPT 4 **Assemblage**

The general principles guiding readers through comic pages, where they seek to build units of structure in as smooth a reading path as possible. These preferences specify that: (1) grouped areas are preferred to non-grouped areas, (2) smooth paths are preferred to broken paths, (3) one should not jump over units, and (4) one should not leave “gaps” in reading.

By following these constraints, readers ultimately form hierarchic relationships between panels and their groupings, organized into horizontal and vertical constituents (Tanaka et al., 2007; Bares, 2008; Cohn, 2013a). These constituents represent the underlying structure that a creator and reader bring to bear on the organization and navigation of a page layout. Figure 3A depicts the ECS for the original *Sinfest* strip described previously. It is fairly simple, three horizontal tiers, with a vertical substructure in the first tier. Figure 3B then alters this layout by rearranging panels, dividing panel 4 into two parts (4.1, 4.2) and adding an “inset” panel (7.2) inside of the more dominant enclosing panel 7.2 (Cohn, 2013a). These changes alter the ECS, but have no impact on the sequence's meaning (the narrative does change though, as discussed below).

## Attentional framing structure

We saw above how altering the framing of panels might change a sequence's layout, but framing might also impact the narrative. For example, framing might determine how many characters appear in a panel, as in Figure 3B in panels 4.1/4.2 or 7.1/7.2: Should two characters at a single narrative state be shown together in a single panel, or should those characters be broken up, each into their own panel? These alterations still do not necessarily change the meaning (semantics) of the sequence, though they do alter the pacing (narrative) and the layout (ECS), and thus aspects of framing seem to operate in between these other structures.

**Figure 4:**
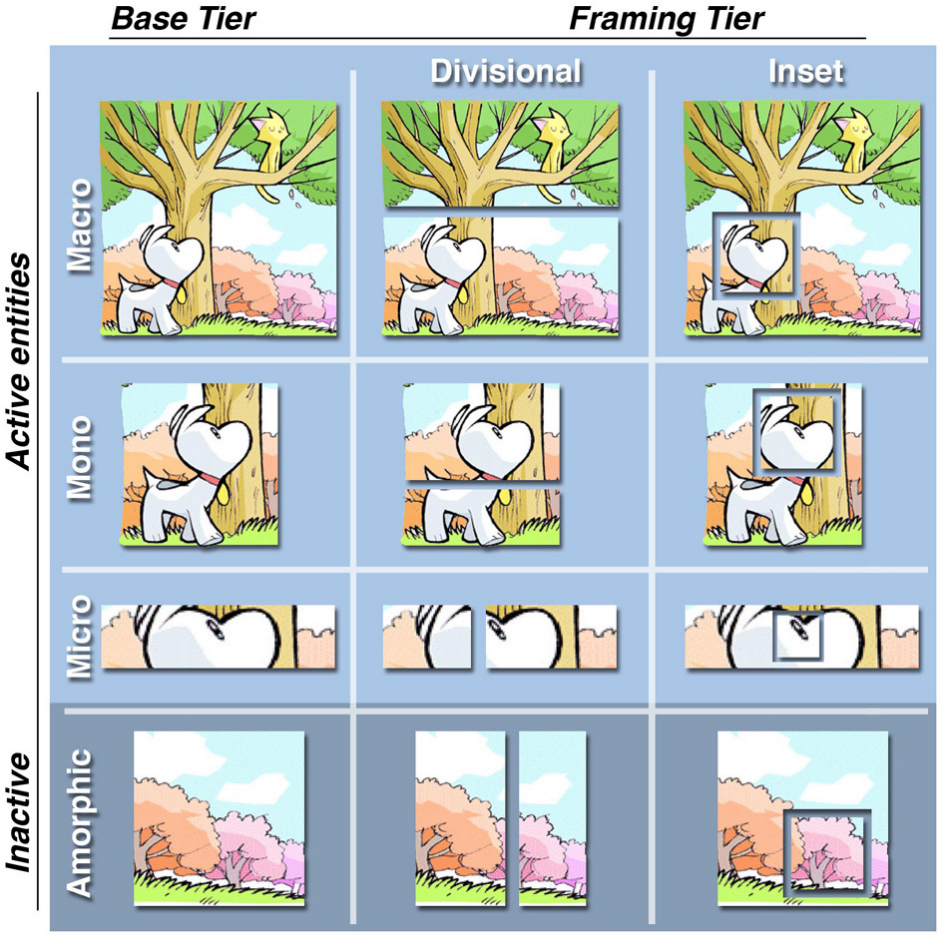
**An “attentional framing matrix” showing how content can be framed in panels across base framing categories and additional modification of aspects of layout**. A *macro* contains multiple active entities engaged in the interaction or situation in the scene. A *mono* contains only a single entity from the scene, while a *micro* depicts less than a single entity, often through a close-up. Finally, *amorphic* panels depict no active entities from the scene—only “inactive” parts of the larger environment or scene. *Divisional* panels break up single images into sub-panels, while *inset* panels are placed within other dominant panels. *Sinfest* and all characters © Tatsuya Ishida.

First, individual panels frame how much information is depicted in a scene. In a sense, the panel borders simulate a “window of attention” that frames only the content an author wants the reader to assimilate. Information that is not directly depicted in panels is either not important or meant to be inferred. Panels therefore act as “attention units” that can be categorized based on how much information they contain, as depicted in the “attentional framing matrix” in Figure 4 (Cohn, 2007, 2013b). In addition, framing intersects with ECS. A single image could be split up into multiple *divisional* panels, where the larger image is recognized because of image constancy, but the component parts individuate certain characters. In addition, *inset* panels may frame information within a larger dominant panel, again to focus attention on that element.

**Figure 5:**
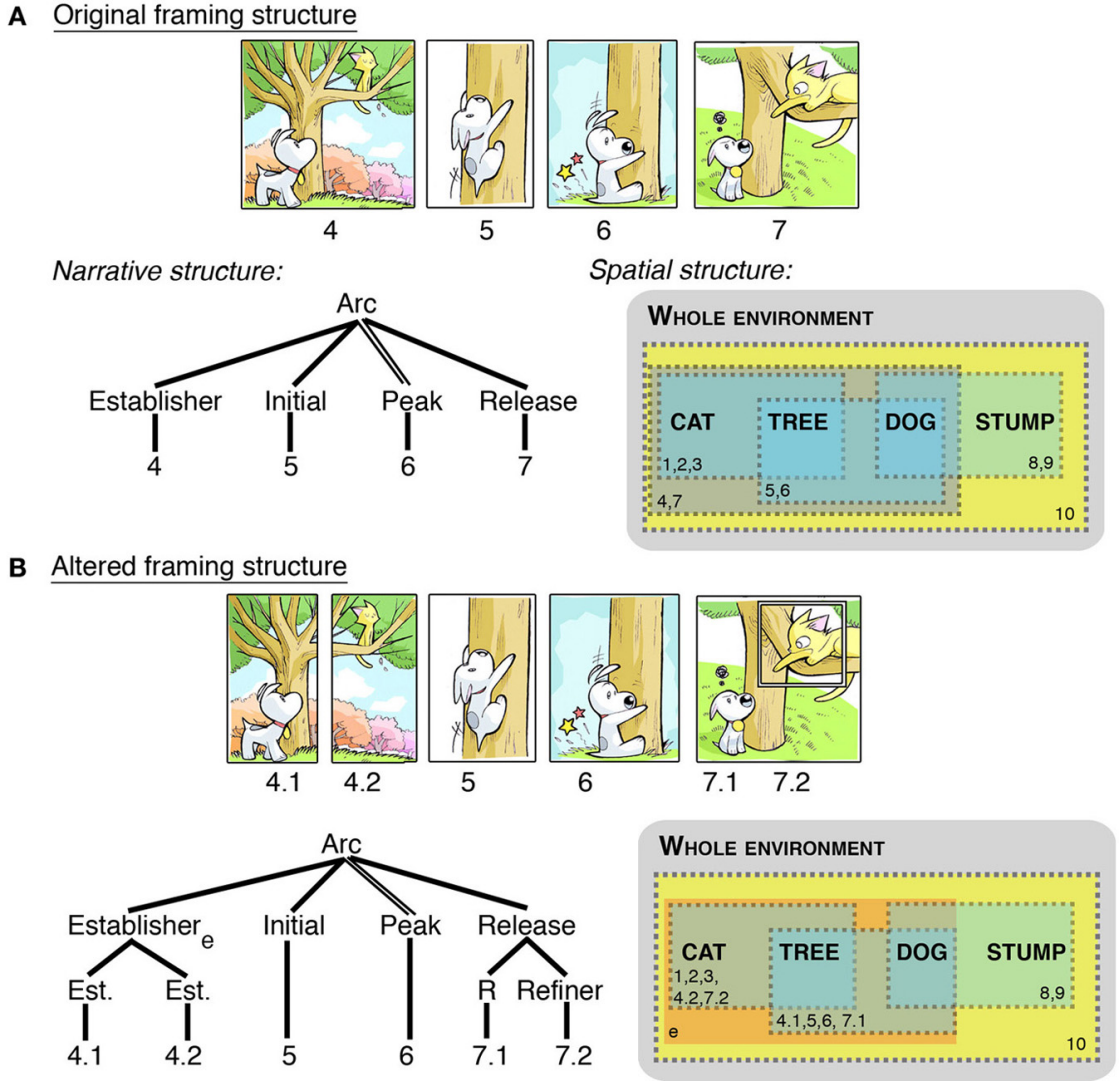
**Visual sequences where alteration of the panel framing changes the narrative structure, along with mapping to a spatial structure**. Note: Extraction of this clause from its context in a larger sequence causes the Establisher to have a local relationship to the rest of the constituent rather than at a higher level, as in Figure 1. **(A)** depicts the original framing, while **(B)** individuates characters, leading to Environmental-Conjunction (notated with subscript “e”). Panel borders added for clarification and emphasis. *Sinfest* and all characters © Tatsuya Ishida.

Figure 5A extracts a sequence from Figure 1A. A spatial representation of this whole scene (cat, dog, tree, stump) illustrates how panels “window” different parts of this overall environment (panels indicated by dotted lines, indexed by panel numbers). Figure 5B alters the original sequence by splitting apart panel 4 (now a divisional), and adding an inset into panel 7. These alterations change the page layout (Figure 3B), but they also change the narrative structure. Dividing panel 4 creates two Establishers conjoined within a larger Establisher constituent, since both panels now play this role. The broader environment that they create (i.e., an environment consisting of both dog and cat together) is now inferred, and is thus depicted in the spatial structure without a dotted border. This “Environmental-Conjunction” is notated with a subscript “e.” In addition, the Release now uses an inset panel to narratively draw focus to an element in a scene—a “Refiner” (Cohn, 2013b). Thus, framing can alter both the narrative and the layout, though the meaning remains largely unchanged.

It is worth noting that how a scene is framed appears to differ across cultures. Corpus analyses suggest that Japanese manga proportionally show less than a whole scene (monos, micros) more often than they show a whole scene (macros), which is different than American comics that tend to show whole scenes more than individuating its component parts (Cohn, 2011; Cohn et al., 2012b). This implies that readers of manga must infer these larger environments more than readers of American comics, where whole scenes are provided outright. Such differences also suggest variance in the way narrative structures are used between cultures.

## Interfaces between narrative and layout

As demonstrated, sequential images involve several structures operating independently of each other, yet all interfacing together. For the example *Sinfest* comic, these connections can be traced between panel numbers across figures. These tree structures are not isomorphic—the constituents in narrative structure do not cleanly align with those from the ECS. For example, in the original layout, the Release of the second narrative constituent (panel 7) starts the third horizontal tier rather than ending a previous tier. Thus, narrative constituent boundaries do not always line up with the boundaries of the physical layout.

This “parallel architecture” of narrative structure and ECS is analogous to the organization of language, where each linguistic substructure (phonology, syntax, semantics) operates with its own principles, yet interfaces with the others to form the whole of linguistic knowledge (Jackendoff, 2002). Because these components are separate, one structure can change while the others remain the same. For example, different layouts can convey the same meaningful content (as in Figure 3B), or the reverse, the same layout could be used for different content.

Future research can better explore the interactions between these structures, such as the mappings that may exist between narrative and layout. Locative information often coincides with the first panel of a page, and suspenseful panels (Initials) often occur at the final panel on a page, thereby inducing a thrilling page turn and subsequent reveal of primary information (Peaks) on the next page (McCloud, 2000). Narrative arcs may alternatively conclude at page borders, thereby using the page layout as a break between constituents. Also, panels that occupy whole “splash pages” are likely to be Peaks—since the large size should echo a climactic moment of the narrative. Inset panels often zoom in on information in a larger panel (“Refiners”), or depict additional characters in the broader scene from the dominant panel (“Environmental-Conjunction”) (see Cohn, 2013b,c). These mappings between narrative and layout could be explored through corpus analyses of comic pages and experimental manipulation.

Beyond these *structural* interfaces, we can also explore how these structures interact in comprehension. Can changes in content force readers to navigate a page in ways that go against their preferred rules? Do readers prefer boundaries between narrative constituents to line up with the boundaries in ECS? What changes in layout might confuse readers about the meaning of the narrative structure? These and other questions can frame future experimentation on the relationship between these structures.

## Conclusion

While concerted scientific research on visual narratives has begun to emerge, these initial forays have shown the advantage of a multilayered approach that balances theoretical modeling, corpus analysis, and empirical experimentation using both behavioral and neurocognitive measures. Altogether, this work has provided evidence for the interactions of narrative, meaning, page layout, and framing, and that familiarity in these structures contributes to a larger fluency in the visual language used in comics.

### Conflict of interest statement

The author declares that the research was conducted in the absence of any commercial or financial relationships that could be construed as a potential conflict of interest.
